# Psychometric Properties of the Hindi Version of the World Health Organization Quality of Life Questionnaire-Older Adults (WHOQOL-OLD) Module in Dehradun, India

**DOI:** 10.7759/cureus.43985

**Published:** 2023-08-23

**Authors:** Kritika Tiwari, Rakesh Kakkar, Pradeep Aggarwal

**Affiliations:** 1 Community Medicine, Army College of Medical Sciences, New Delhi, IND; 2 Community and Family Medicine, All India Institute of Medical Sciences, Bathinda, Bathinda, IND; 3 Community and Family Medicine, All India Institute of Medical Sciences, Rishikesh, Rishikesh, IND

**Keywords:** whoqol-old, reliability, validity, psychometric properties, quality of life, india, factor analysis, elderly

## Abstract

Background: The perception of quality of life (QOL) is a subjective concept; however, attempts have been made to discern the key points and to give a metric view of this concept. The World Health Organization Quality of Life Questionnaire-Older Adults (WHOQOL-OLD) module is an international and cross-cultural tool that quantifies the QOL in older adults.

Objectives: This study aimed to assess the psychometric properties of the Hindi version of the WHOQOL-OLD module by calculating its reliability and validity among the elderly residing in the Dehradun district of India.

Methodology: This cross-sectional study was conducted among 440 elderlies from the rural and urban areas of Dehradun by using the Hindi version of the WHOQOL-OLD questionnaire. The mean and standard deviations were calculated for QOL scores. Reliability was checked by calculating Cronbach’s alpha (α), and factor analysis was done for the validity of the questionnaire.

Results: Mean (±SD) for total QOL score was 54.3 (±9.3). The death and dying facet had the maximum mean score, whereas the minimum mean score was calculated in the autonomy facet. Cronbach’s alpha reliability coefficient for the overall QOL score was calculated as 0.86 which shows good internal consistency of the items in the questionnaire. To measure the construct validity, exploratory factor analysis (EFA) by principal components analysis (PCA) was performed on the 24 items of the WHOQOL-OLD module, and a six-factor model was identified. Satisfactory goodness-of-fit statistics were found on the confirmatory factor analysis (CFA).

Conclusion: QOL is a multidimensional concept. The Hindi version of the WHOQOL-OLD module is reliable and valid. QOL in the elderly population can be measured by using the WHOQOL-OLD module in India.

## Introduction

Aging is a complex dynamic process and is inevitable. The focus of modern geriatrics is to add years to life and health to years. This has instigated research on quality of life (QOL) which has now assumed a pivotal position in geriatric research [1,2]. There is a plethora of views on QOL. It is a labyrinthine, diffuse, and multidimensional concept that has a colossal impact on research and practice. The definition of QOL propounded by WHO has been widely accepted which revolves around individuals’ subjective perception of their locus in life with respect to health, environment, relationship, expectations, ambitions, and psychology [3,4]. The perception of QOL is a subjective concept; however, attempts have been made to discern the key points and to give a metric view of this concept. The incongruence and diversity of the determinants of QOL in old age advocate a different method of assessment from the general population. This assessment should include multiple domains. However, the direct and precise measurement of QOL is not executable. It can only be measured indirectly with the aim of measuring it as close to the real value as possible with a minimum of random error. Developing a conceptual framework and assessing the QOL precisely among the geriatric population obligates empathy and a need to visualize things from their perspective by understanding their perceptions of QOL [5].

The definition of QOL is wide and subjective rather than specific and objective. Due to that complex relationship, the measurement of QOL is quite difficult and requires necessary instruments. Thus, researchers have developed useful tools for measuring QOL [6]. There are several methods/tools to measure QOL. Some of the most important tools have been developed by WHO to measure QOL. There are specific areas of QOL that may be more important in older adults. For this, the World Health Organization Quality of Life Questionnaire-Older Adults (WHOQOL-OLD) questionnaire has been developed by the WHO. This module was subjected to various steps for its formulation and validation cross-culturally [7]. It has been translated into many languages including Hindi which is a widely spoken and understood language in India.

The objective of this study was to assess the psychometric properties of the Hindi version of the WHOQOL-OLD module by calculating its reliability and validity among the elderly residing in the Dehradun district of India.

## Materials and methods

Study design and study area

The present research was a cross-sectional study conducted among the geriatric population of the rural and urban areas of the Dehradun district of India.

Sample size

The minimum sample was calculated to be 220 by using the formula (1.96)^2^σ^2^/l^2^ and 10% as the non-response rate. The following values were put in the abovementioned formula: σ - standard deviation = 10.88 [8] and l - tolerable error = 1.5%. Ninety-five percent was taken as the confidence interval. The study was conducted in rural as well as urban areas. Hence, the final sample size was 440.

Inclusion criteria

Subjects with age ≥60 years who were residing in that area for at least five years and gave their written consent were included in this study.

Exclusion criteria

The elderly who were not willing to participate in the study or were critically ill were excluded.

Sampling technique

**Figure 1:**
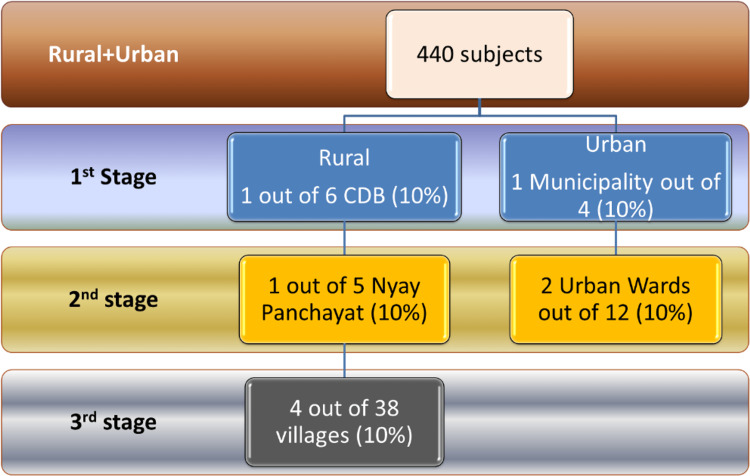
Sampling technique for rural and urban areas

Multistage random sampling was done in three stages for rural areas and two stages for urban areas. In the first stage, one community development block (CDB) was selected randomly out of six for rural areas, and one municipality was selected randomly out of four for urban areas. In the second stage, one nyay panchayat was selected randomly out of a total of the five nyay panchayats of the previously selected CDB, and two urban wards were selected randomly out of the 12 urban wards from the previously selected municipality. In the third stage, four villages were selected randomly out of the total 38 villages in that nyay panchayat (Figure 1).

Selection of subjects

Equal samples, i.e., 55 subjects from each of the four villages and 110 from each of the two wards were included in the study. All the elderly in the selected villages and wards constituted the sampling frame. A house-to-house survey was done, and eligible geriatric people were selected by consecutive sampling till the accomplishment of the sample size. If more than one eligible elderly was present in a family, only one was selected by lottery method.

Study tools

Socio-demographic details like age, gender, marital status, education, and financial status were collected by questionnaire method. The Hindi version of the WHOQOL-OLD questionnaire was used to assess QOL. There are six facets with a total of 24 Likert-scaled items in this questionnaire: The first facet is SAB (sensory abilities) which assesses sensory functioning and the impact of the loss of sensory abilities on QOL. The second facet, AUT (autonomy), assesses the ability to take own decisions and independence in old age. The third facet is PPF (past, present, and future activities) which describes satisfaction with achievements in life and things looking forward to. SOP (social participation) is the fourth facet and it delineates participation in community activities. The fifth facet is DAD (death and dying) which deals with the worries, fears, and concerns about the end stage of life, i.e., death. The sixth facet, INT (intimacy), focuses on intimate and personal relationships. Each of these six facets has four items on the 5-point Likert scale; thus, for all facets, the score of possible values can range from 4 to 20, provided all items of a facet have been completed. The values of the 24 single items/scores of the six facets, when combined, produce a general or overall score for QOL in older adults [9].

Ethical consideration

Permission from the WHO to use the questionnaire and approval from the Institutional Ethical Committee of the Himalayan Institute of Medical Sciences (SRHU/HIMS/ETHICS/2016/103) was taken before starting the study. All the subjects agreed to participate in the study by written informed consent.

Statistical analysis

SPSS Statistics version 22 (IBM Corp. Released 2013. IBM SPSS Statistics for Windows, Version 22.0. Armonk, NY: IBM Corp.) and IBM SPSS Amos (IBM Corp., Armonk, NY) were used for data analysis. The mean and standard deviations were calculated for QOL scores. Reliability was checked by calculating Cronbach’s alpha (α), and factor analysis was done for the validity of the Hindi version of the WHOQOL-OLD questionnaire.

## Results

The gender-wise distribution of the socio-demographic characteristics of the study population is shown in Table 1. The maximum number of study participants (both males and females) were in the age group 66-75 years. About 64.5% of the elderly were living with their spouse. Most of the males were literate, but the literacy rate was less among the female population with more than 60% illiterate women. More than 50% of males were financially independent, whereas most of the females were financially dependent on their family members. Almost equal percentages of male and female elderly had some source of income and were partially dependent on their family members financially.

**Table 1:** Gender-wise distribution of the socio-demographic characteristics of the study population

Socio-demographic variables	Gender		
	Male (n=218)	Female (n=222)	Total (n=440)
Age (years)			
60-65	75 (34.4%)	86 (38.7%)	161 (36.6%)
66-75	88 (40.4%)	94 (42.3%)	182 (41.4%)
76-99	55 (25.2%)	42 (18.9%)	97 (22.0%)
Marital status			
Married	152 (69.7%)	132 (59.5%)	284 (64.5%)
Widow/separated/divorced	66 (30.3%)	90 (40.5%)	156 (35.5%)
Education			
Literate	160 (73.4%)	83 (37.4%)	243 (55.2%)
Illiterate	58 (26.6%)	139 (62.6%)	197 (44.4%)
Financial status			
Dependent	44 (20.2%)	138 (62.2%)	182 (41.4%)
Partially dependent	49 (22.5%)	59 (26.6%)	108 (24.5%)
Independent	125 (57.3%)	25 (11.3%)	150 (34.1%)

The descriptive statistics of all six facets and total QOL scores among the study population were calculated and represented as mean and standard deviation (Table 2). The mean (±SD) for the total QOL score was 54.3 (±9.3). The death and dying facet had the maximum mean score, whereas the minimum mean score was calculated in the autonomy facet.

**Table 2:** Descriptive statistics of facet and total QOL scores and reliability of the Hindi version of WHOQOL-OLD among the study population QOL: quality of life, SAB: sensory abilities, AUT: autonomy, PPF: past, present, and future activities, SOP: social participation, DAD: death and dying, INT: intimacy

QOL facets	Mean (±SD)	Cronbach’s alpha (α)
SAB	61.5 (±14.7)	0.76
AUT	42.8 (±14.5)	0.61
PPF	49.0 (±12.8)	0.70
SOP	53.2 (±14.2)	0.78
DAD	73.5 (±19.2)	0.89
INT	45.7 (±14.6)	0.90
Total (overall)	54.3 (±9.3)	0.86

To check the reliability of the Hindi version of the WHOQOL-OLD questionnaire, Cronbach’s alpha (α) was calculated. The value of Cronbach’s alpha reliability coefficient was acceptable for each facet, and its value for the overall QOL score was calculated as 0.86 which shows good internal consistency of the items in the questionnaire (Table 2).

To measure the construct validity, exploratory factor analysis (EFA) by principal components analysis (PCA) was performed on the 24 items of WHOQOL-OLD by using the SPSS Statistics software. Kaiser-Meyer-Olkin (KMO) test was 0.837 which exceeds the threshold of 0.60, and Bartlett’s test was statistically significant (p<0.0001). These values support the factorability of the correlation matrix and confirm that the use of factor analysis was appropriate.

**Figure 2:**
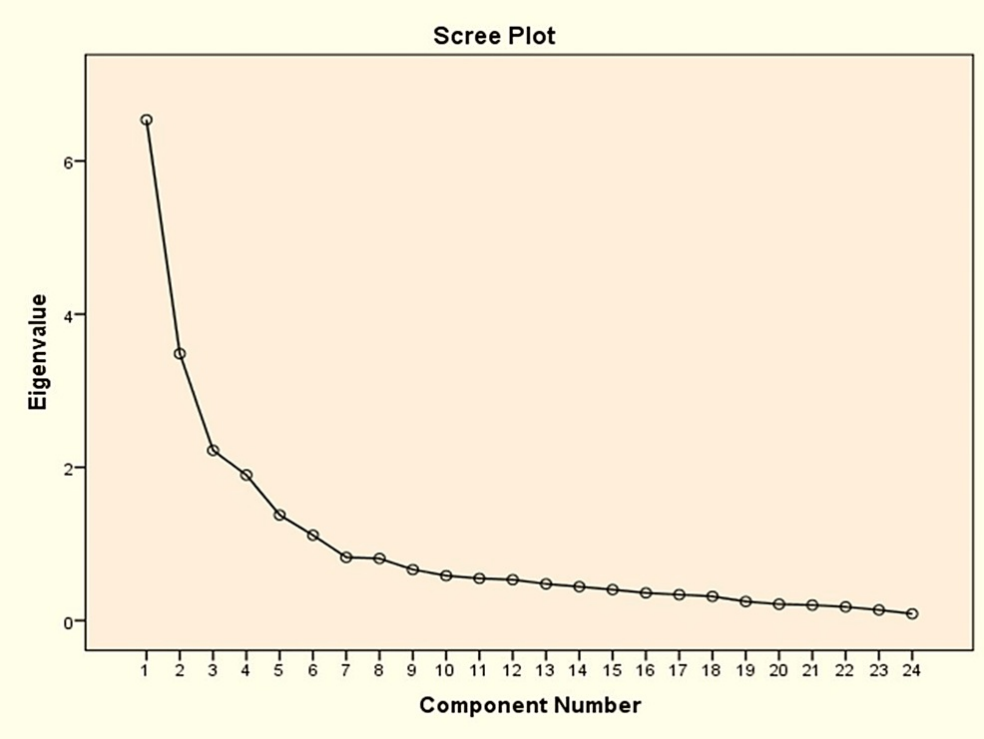
Scree plot

The results for the number of factors are depicted in Table 3 and Figure 2. On PCA, six components were identified which had Eigenvalues greater than one. The scree plot depicts the number of components on the X-axis and Eigenvalues on the Y-axis. About 69.4% of the variance in QOL between the subjects was explained by these six components together. The six-factor model was confirmed by parallel analysis.

**Table 3:** PCA (total variance explained)

Components	1	2	3	4	5	6
% of variance	14.4	13.4	13.0	11.9	9.8	6.9
Cumulative %	14.4	27.8	40.8	52.7	62.5	69.4

**Figure 3:**
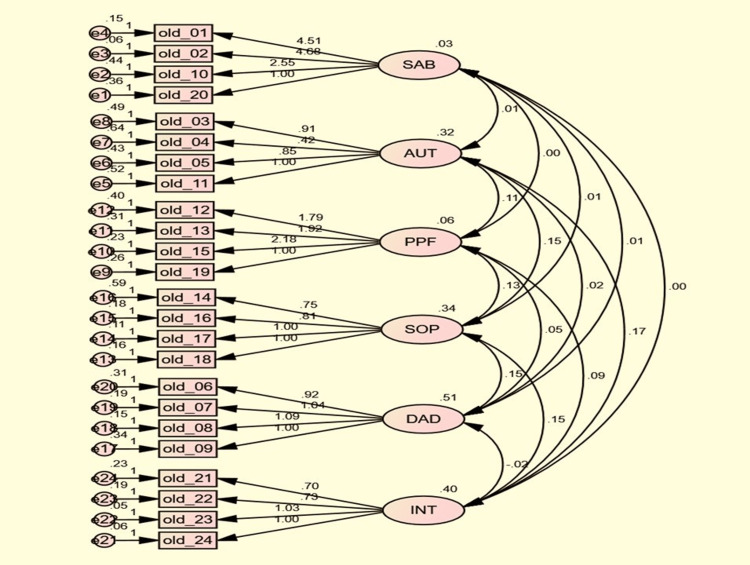
Path diagram of CFA SAB: sensory abilities, AUT: autonomy, PPF: past, present, and future activities, SOP: social participation, DAD: death and dying, INT: intimacy

To see whether the data from this study fit the hypothesized model of WHOQOL-OLD, confirmatory factor analysis (CFA) was done by IBM SPSS Amos software. The path diagram is represented in Figure 3. The ovals in the figures are the six factors corresponding to the six facets of the WHOQOL-OLD questionnaire (SAB, AUT, PPF, SOP, DAD, and INT). The double-headed arrows between these factors represent the covariances. The variances of these factors are represented above the ovals. The rectangles represent the items under the particular factor e.g. old_01 is the first question of the questionnaire and it lies under the SAB facet. The figure also depicts the factor loadings or regression slopes and the error terms with residual variances. Goodness-of-fit statistics for the model were calculated. A non-significant Chi-square test, a higher value of the comparative fit index, and the root mean square error of approximation value of <0.05 show that the model fits the data adequately.

## Discussion

The psychometric properties of the Hindi version of the WHOQOL-OLD module were assessed in this study among 440 subjects above 60 years of age. The original version of WHOQOL-OLD has been translated into many languages, and studies have been performed to check the psychometric properties of these translated versions. This study recruited an almost equal number of male and female elderly participants. Most of the studies conducted in other countries had less percentage of male participants [9-14], but the study on the Dutch WHOQOL-OLD had more male participants [15]. About 64.5% of the study subjects of this research were living with a spouse which was similar to the subjects in some other studies [10,11,13], whereas this percentage was less [9,12,14] and more [13,15] in other studies. A large percentage (44.4%) of the elderly were illiterate in our study, and this percentage was the maximum among other studies done in this context in different countries [10,11,13,14]. Out of the illiterate subjects, the maximum number was of female participants in our study. Only 34.1% of the elderly were financially independent in our study like a study in Ghana by Anum et al. on 353 elderly [12], whereas this percentage was reversed in a study in Nigeria [10]. The differences in these socio-demographic indicators between the studies in different countries of the world could lead to differences in the results of QOL.

The total/overall score of QOL by using the WHOQOL-OLD questionnaire in the Hindi language was 54.3 (±9.3) among the subjects of this study. This score was the least among other similar international studies [10,13,15-17] where the range of scores was from 77.72 (±10.41) to 95.66 (±18.56), but the score of our study was comparable to a study done in Turkey where the score was 56.02 (±11.6) [18]. A comparative study between Brazil and India by Figueira et al. found lesser QOL scores in both Brazilian and Indian elderly as compared to this study [19]. These differences could arise because of different cultural set-ups and different value systems and beliefs which affect the perception of QOL. The death and dying facet had the maximum QOL score, whereas the autonomy facet had the minimum QOL score in this study. This could be because the Indian elderly accept death as inevitable, but they lack autonomy in their life. On the contrary, the death and dying facet had the least score in a study conducted among the Dutch elderly [15]. Intimacy had the maximum score in many studies [10,12,16,18], but this facet had the second lowest score in our study which indicates a lack of close company in older age in this subset of Indian elderly. In a study by Robbert et al. [15], more than 70% of the elderly were living with their spouse, but still the intimacy facet was the least scored one.

Good internal consistency was found among the items in this study (Cronbach’s alpha 0.86) which was similar to the results of other studies testing the reliability of WHOQOL-OLD [13-15,18,20,21]. Cronbach’s alpha was found to be >0.9 in studies done in Nigeria and Ghana [10,12]. In our study, Cronbach’s alpha was maximum in the intimacy facet and minimum in the autonomy one which is similar to a study done in Indonesia [11]. The intimacy facet had the highest value of Cronbach’s alpha in some other studies too [9,15]. The autonomy facet had the lowest Cronbach’s alpha among the Turkish elderly [18].

In EFA, this study proposed a six-factor model with 69.4% of the variance in QOL between the subjects being explained by these six components together. A 62.95% variance was explained by the six-factor model in a Chinese study [20]. The goodness-of fit-statistics were found to be satisfactory in our study for the six-factor model like other studies [9,13,15,21,22]. A Nigerian study proposed a four-factor model [10], whereas a study by Erhan et al. in Turkey proposed a five-factor model with "PPF" and "SOP" as a single factor [16]. Some overlapping between item scales was observed in a study by Sultan et al. [18]. A South African study also proposed a shorter version of WHOQOL-OLD [22]. A recent study, done on the psychometric properties of the Persian version of WHOQOL-OLD by Ghahremani et al. in 2023 [23], found this version to be reliable and valid with the proposed six-domain structure. A cut-off value for the QOL score (71.5) was also proposed in this study between good and poor QOL.

The WHO questionnaire for assessing QOL in older people has been found to be valid in many countries in various languages and our study found the Hindi language version of this questionnaire to be valid too for the Indian elderly population studied.

Recommendations

Research on the less explored topic of QOL among the elderly by using the WHOQOL-OLD module is recommended by the authors to measure the QOL in this vulnerable population so that the areas of poor QOL can be addressed.

Limitations of the study

This study was a small-scale cross-sectional study in one district of Uttarakhand (India).

## Conclusions

The present study was conducted among elderlies residing in rural and urban areas of the Dehradun district of Uttarakhand (India), using the Hindi version of the WHOQOL-OLD questionnaire. The psychometric properties of this questionnaire were assessed by calculating the reliability and validity through Cronbach's alpha and factor analysis. The Hindi version of the WHOQOL-OLD questionnaire was found to be reliable and valid. Six facets were identified in the Hindi version of this questionnaire which supports the WHO's six-factor model. This model, however, could not explain the entire variance in QOL which opens up the scope of further research in this area. Hence, QOL is a multidimensional and crucial concept in the context of the elderly population. QOL in the elderly population can be measured by using the WHOQOL-OLD module in India.
